# Driving innovation in health care: exploring the impact of ambidextrous leadership on creative performance among frontline health professionals in Norway

**DOI:** 10.1186/s12913-024-10641-9

**Published:** 2024-03-02

**Authors:** Barbara Rebecca Mutonyi, Manel González-Piñero, Terje Slåtten, Gudbrand Lien

**Affiliations:** 1 School of Economics, Innovation and Technology, Kristiania University College, Oslo, Norway; 2 Department of Economics, Faculty of Economics and Business, University of Barcelona, John M. Keynes 1-11, 08034 Barcelona, Spain; 3 Research Centre for Biomedical Engineering, Technical University of Catalonia, Diagonal 647, 08028 Barcelona, Spain; 4 Inland School of Business and Social Science, Inland Norway University of Applied Sciences, Campus Lillehammer, 2604 Lillehammer, Norway

**Keywords:** Creative performance, Ambidextrous leadership, Relationship learning, Learning orientation, Health professionals

## Abstract

**Background:**

In recent years, there has been an increasing focus on enhancing frontline health professionals’ ability to think and act innovatively, also known as their creative performance. However, previous research has had two limitations. First, only a few leadership styles and their associations with this capability have been examined. Second, there has been a lack of research on identifying potential process mediators and examining their role in the relationship between leadership styles and the professionals’ capability. To address this knowledge gap, our study investigates the impact of ambidextrous leadership, a relatively new leadership style, on frontline health professionals’ creative performance. Additionally, we explore whether frontline health professionals’ learning orientation (an individual factor) and relationship learning (an organizational factor) act as process mediators in this association. No previous research has focused on these relationships. Thus, the study offers a unique contribution to health services research.

**Methods:**

This is a cross-sectional study with a convenience sample of *N =* 258 health professionals in nine Norwegian municipalities. The results of this study were analyzed using PLS-SEM with SmartPLS 3 software. The study examined both direct and indirect relationships through bootstrapping.

**Results:**

The results reveal a positive link between health professionals’ creative performance and ambidextrous leadership $(\beta$ = 0.224). Both relationship learning and learning orientation were found to operate as complementary process-mediating factors between health professionals’ creative performance and ambidextrous leadership. The strength of the two individual relationships that constitute the process-mediating factors indicates that ambidextrous leadership has a stronger impact on relationship learning than on learning orientation $(\beta$ = 0.504 versus $\beta$ = 0.276). However, when we examined the individual associations between the two factors and creative performance, the strength of the relationships was quite different. The findings reveal that learning orientation is significantly more positively associated with creative performance than relationship learning $(\beta$ = 0.302 versus $\beta$ = 0.163). Ambidextrous leadership, learning orientation, and relationship learning explain 26% (*R*^2^ = 0.262) of the variance in professionals’ creative performance.

**Conclusions:**

This study suggests that ambidextrous leadership can facilitate health professionals’ creative performance directly and indirectly through the two process-mediating factors: relationship learning and learning orientation. Thus, a practical implication is the importance for health service organizations of clear awareness of the numerous advantages of having leaders who actively practice an ambidextrous leadership style.

## Background

The ever-evolving competitive landscape in today’s business environment has heightened the demand for organizational flexibility and performance. Successful teams within companies are characterized by the compatibility and complementarity of roles played by their members [1–3]. Particularly in larger organizations, where innovation thrives on diverse skill sets and perspectives, synergy among team members is a cornerstone of success.

### Innovation in the context of health care organizations

Innovation is not a luxury but a necessity for organizations aspiring to maintain a competitive edge and ensure long-term viability [4]. The competitive environment continually underscores the importance of innovation, as it creates novel value that outpaces competitors in a dynamic market [4, 5].

The health care sector, the focal point of this study, is no exception to the imperative of innovation. Innovation transcends the introduction of new services or technologies; it encompasses a shift in perspective that positions patients as active consumers rather than passive users [6, 7]. This patient-centric approach is essential to improving organizational service effectiveness, emphasizing a market-oriented business culture, for two primary reasons. First, it enables organizations to gather crucial information about their target customers’ needs and their competitors’ capabilities consistently. This customer-centric focus aligns organizations more closely with their customers’ requirements. Second, this approach helps organizations create substantial customer value [8].

In the context of health care, patients constitute the primary customer base. Thus, health care service providers must be able to adapt their services in a manner that aligns with the realities of patients’ experiences [9–11]. Frontline health professionals who directly engage with patients occupy a unique vantage point to observe, identify, and address service gaps. Their proximity to patients empowers them to think creatively and act innovatively, ultimately enhancing the overall patient experience [12]. This rationale resonates with Hewko’s assertion that “frontline staff, in particular, are well-suited to develop beneficial innovations” [13]. Similarly, Kim and Park [14] underscore the pivotal role of individuals in driving innovation in organizations. As a result, understanding the factors that facilitate creative thinking and innovative action among frontline health professionals has become paramount.

### The role of leadership and innovation

While prior research has predominantly studied innovation from an organizational perspective in the health care domain [6, 15], there has been limited empirical examination of individual employees’ capacity for innovation [6]. These studies have focused on a variety of factors, such as the role of employee empowerment [1], organizational culture, leadership support, and organizational attractiveness [5], employees’ level of motivation and stress [7], and whether organizational vision integration and employee psychological capital [11] are associated with an employee’s capability to innovate. However, the number of previous studies is limited.

Oppi, Bagheri [16] highlighted the scarcity of research on factors facilitating health care innovation, particularly among frontline health professionals. This gap calls for further investigation to broaden the list of facilitating factors and identify new ones associated with creative performance (CP).

The role of leadership in innovation has drawn attention, with Zaccaro, Dubrow [17] innate traits and Northouse [18] suggesting the importance of situational factors. Kim and Beehr [19] research supports this by linking positive work cultures to leadership empowerment strategies. Genetics, environment, and development collectively influence leadership, as seen in studies on twins by Arvey, Rotundo [20].

Ambidextrous leadership (AL) in this study refers to leadership practices that have “the ability to foster both explorative and exploitative behaviors in followers by increasing or reducing variance in their behavior and flexible switching between those behaviors” [21]. Recently, AL has been suggested as a promising style that facilitates health professionals’ capability to think creatively and act innovatively [22]. However, according to Slåtten, Mutonyi [23], it is evident of the scarcity of research, in health services research, empirically examining the impact of AL on factors associated with innovation.

The objectives of this study are threefold:

1. **Research Question**: This study seeks to answer the question: What is the relationship between AL and health professionals’ CP?
2. **Research Gap and Importance**. The paper has three aims based on the knowledge gaps identified in previous research. First, as indicated above, it examines how the AL style can facilitate frontline health professionals thinking creatively and acting innovatively, which this study calls CP. Second, the study aims to explore empirically how two different process mediators operate in the relationship between AL and CP. Specifically, building on ideas in previous health services research [23], the study explores how health professionals’ learning orientation (LO: an individual factor) and relationship learning (RL: an organizational factor) act as process mediators between AL and CP. Third, to address the second aim, it reveals how the two process mediators in this study (LO and RL) are individually associated with CP and AL.
3. **Potential Contributions**. This study offers a unique contribution to health services research because, to the authors’ knowledge, no previous research has focused on these aspects in a health care setting. By delving into the factors influencing the CP of frontline health professionals, the study responds directly to a recent call for more research revealing the association between AL and employees’ innovative behavior or CP [11, 23]. In addition, it responds to a (recent) call for more research on process mediators associated with AL. In previous research on the process, Usman, Ghani [24] state: “It is mainly unknown through which processes AL affects IWB (innovative work behavior).”

**Fig. 1:**
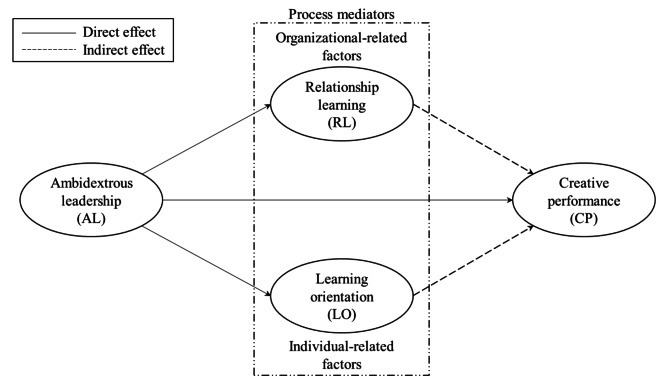
Conceptual model

The purpose of this paper is shown in Fig. 1. AL is assumed to be associated with health professionals’ CP. In addition, it is also assumed that two types of process mediators operate between AL and CP. Both are located within the dotted lines shown in the middle of Fig. 1. The dotted lines indicate that this study restricts the number of process mediators to two.

The two process mediators are (i) RL and (ii) LO. The two process mediators are distinct and represent different types. RL represents an organizational factor, while LO represents an individual factor. As indicated in Fig. 1, it is assumed that AL is associated with health professionals via two paths. The first is direct; the second is indirect and works through the two process mediators.

### Review of the literature

The literature review in this section identifies the concepts and links shown in Fig. 1.

#### Creative performance (CP)

According to Patterson and Zibarras [25], there is an increased need for employees to produce and implement new ideas to improve work efficiency. Therefore, individual health professionals with different capabilities and interests must be capable of generating ideas, being creative, and implementing these (creative) ideas in their work practices. This may be labeled innovative behavior.

Consequently, it is insufficient for health professionals to be capable of thinking creatively. They must put these ideas into real action (innovative behavior) and improve their work performance. Sometimes, innovations that have not previously been mapped encounter massive structural barriers and resistance to change called a “lock-in” of the health care system [26, 27]. The structures, relations, and processes that determine the path of an innovation from invention are relevant because both creativity and innovative behavior are present in the concept of CP. Specifically, CP in this study is centered on individual frontline health professionals’ work practices related to and manifested through their work role performance. In line with previous research, CP reflects a combination of both cognitive elements (creativity) and behavioral elements (innovative behavior) [11].

Managing creativity to accelerate and improve innovation is the key management challenge companies will face in the coming years in an environment of ever-increasing complexity [28]. The emergence of new ideas is a necessary yet insufficient condition for innovation. These ideas are mostly black boxes in innovation theories and must be addressed as processes [29]. Regarding this closeness and integration of creativity and innovation, Anderson, Potočnik [30] noted that both concepts at work concern processes, outcomes, and products to develop and introduce new or improved ways of doing things. Creativity refers to generating ideas, while innovation is subsequent implementation to improve procedures, practices, services, or products [31]. In line with Anderson, Potočnik [30], it is important to note that creativity and innovation can occur at the individual employee level. Patterson and Zibarras [25] supported the idea of closeness between creativity and innovation, as the two constructs often overlap. For instance, Anderson, Potočnik [30] noted that the boundaries between creativity and innovation are unclear. This overlap is (most likely) a natural result of creativity being embedded in and part of innovation, whether incremental or radical. Thus, innovation, especially from a frontline perspective (as in this study), is a tangible aspect and a manifestation of creativity.

Consequently, creativity and innovation can be combined into one concept, CP, that embraces two key sources or ingredients of potentially positive changes or improvements in work performance. Therefore, in line with Slåtten, Mutonyi [11], frontline health professionals’ CP in this study is service embodied in the employees’ work roles.

#### Ambidextrous leadership (AL)

Leadership is a must in this global competitive work environment. Leadership is a process by which a person influences others to accomplish an objective and directs the organization cohesively and coherently [32]. This definition is aligned with that of Northouse [33], who argued that leadership at work is a process whereby a leader uses knowledge and skills to stimulate employees to achieve a common goal.

Leadership is key to compassionate, high-quality, patient-centered care [34]. The relationship between the approach of leaders (or their leadership style) and the context in which they operate is seen as important for influencing employees’ attitudes and/or behavior. However, as discussed in the Introduction and shown in Fig. 1, we focus on a single leadership style, AL.

Ambidexterity is explained by the Merriam-Webster [35] dictionary as using both hands with equal ease or dexterity. Consequently, ‘ambidextrous’ originally described people with the skill or capability to use both hands equally [21]. Applying the term to a leadership style and practices indicates that the leader is skilled and capable of combining two different and distinct leadership styles with equal ease.

As mentioned above, AL is understood to foster both explorative and exploitative behavior among employees [21]. This definition emphasizes four elements central to its value in a workplace setting. These four central elements are briefly explained below.

First, the definition pinpoints the goal and purpose of practicing AL. As the definition suggests, it concerns fostering explorative and exploitative behaviors among employees. These two types of behavior are diametric. Explorative behavior is exploring new directions that seem unconventional and risky [36–38]. In contrast, exploitative behavior focuses on efficiency through goal attainment while avoiding risk and errors [36–38]. As such, AL fosters both explorative and exploitative behavior among employees [21].

Second, AL suggests what leaders should do, through their leadership practices, to stimulate and convince followers to pursue explorative and exploitative behavior. An ambidextrous leader can achieve this through leadership behavior that is both open and closed. For example, open leadership allows employees to accomplish tasks while encouraging independent thinking, action, and learning from errors [21]. In contrast, closed leadership often includes monitoring and enforcing compliance, sanctioning errors, and working through established routines [21]. Therefore, Rosing, Frese [21] recommended that open and closed leadership behaviors be complementary and combined to achieve efficiency in working toward a common goal.

Third, AL advocates the importance of flexibility in switching between and adjusting opening and closing leadership according to the situation or task [21]. Therefore, Rosing, Frese [21] recommended that AL be adjusted, combined, and matched appropriately to motivate professionals to perform specific tasks in the desired way (e.g., to strengthen their CP). AL theory states that opening and closing leader behaviors moderate each other’s effects on innovation, such that CP is highest when both opening and closing leader behaviors are prevalent [39, 40]. With AL, the use of opening and closing behaviors will vary across time, situation, and tasks. This implies that predicting and predetermining the optimal combination of the two leadership behaviors in advance is difficult. Thus, both types of leadership behavior need to be present for a task or situation [21].

Fourth and last, AL signals the ability of a leader. Ability is the possession of the means or skill to do something. This implies that the extent to which a leader can practice or use AL will vary along a continuum from low to high AL ability. Therefore, AL is heterogeneously distributed among leaders within and between organizations. An implication of this is that when AL is practiced positively and appropriately, it could serve as a potential competitive advantage for an organization (e.g., innovativeness).

In summary, the four central elements highlight four key aspects of AL and its value at work: i), the goal and purpose of practicing AL, ii), what leaders should do to promote AL, iii), the flexibility of AL for a given situation or task, and iv), variation in the use of AL. Figure 1 indicates that practicing AL impacts the CP of health professionals. As mentioned above, CP involves both idea generation (creativity) and idea implementation (behavior) to improve work practices [25]. These two elements make CP an intricate component as it embraces both thinking creatively and innovative action. In addition, as discussed above, the four aspects that characterize AL are especially useful for CP. Consequently, there is a need for a leadership style that enables both elements of CP (creativity and innovative behavior).

Previous research has shown that AL is a suitable catalyst for innovation. Essentially, the theory of AL was originally developed specifically to capture the complexity of innovation with a leadership theory to match this complexity [21, 30, 41].

The preference for Ambidextrous Leadership (AL) over other styles, particularly transformational leadership, stems from its unique and targeted attributes that directly contribute to enhancing Creative Performance (CP) [21, 40]. Unlike transformational leadership, which broadly encourages learning through intellectual stimulation [42], AL offers a more tailored and effective approach to driving innovation within a team: (1) AL stands out for its focus on specific, task-related leader behaviors that cater directly to the intricacies of the innovation process [21, 40]. In contrast, transformational leadership, while promoting intellectual stimulation, tends to address general motivation rather than providing concrete advice for task performance associated with creativity. (2) AL’s model takes into account the situational requirements of both creativity and implementation, providing a strategic basis for leaders in the innovation process [21, 40]. This alignment ensures that leader behaviors are effective in fostering both the creative thinking and the practical implementation required for CP. (3) Empirical evidence, such as the findings from Gerlach, Hundeling [43], reinforces the superiority of AL in driving innovation performance. The study highlights AL’s positive relationship with innovation outcomes [43], setting it apart from other leadership styles, including transformational leadership.

In essence, AL’s effectiveness lies in its ability to bridge the gap between creativity and implementation, offering a more direct and tailored approach to enhancing CP within a team. The effectiveness is further underscored by leaders’ individual decisions on when and how to use opening and closing behavior, making it a positive driver of CP [42]. The AL use of opening behavior to encourage individual employees to think differently [24] directly stimulates the creative (cognitive thinking) element of CP. However, opening behavior is not limited to stimulating creative thinking but is also designed to encourage employees to implement their creative ideas while taking risks [24]. Opening behavior thus stimulates the behavioral element of CP, acting innovatively. Consequently, opening behavior is positively associated with both elements of CP. However, there is a limitation on the degree to which AL should stimulate opening behavior. In line with ambidexterity theory, opening behavior should be combined with closing behavior, such as establishing routines, monitoring, and taking corrective action [24]. Opening or closing behaviors depends on time, situation, and task. They should be adapted to each situation [21]. Therefore, a leader must correctly understand and adopt the appropriate opening and closing behavior. Usman, Ghani [24] added that the role of an ambidextrous leader is crucial, as leaders must decide when to switch between the two behaviors in a given situation.

This study does not specifically consider the balance between the opening and closing behaviors associated with CP. It focuses on whether the combination of opening and closing behavior can promote CP among health professionals. To the authors’ knowledge, this is a pioneering empirical study indicating a link between AL and CP in health services research. However, research in other domains has identified a positive association between the two. Studies undertaken in different countries and contexts strongly support a positive relationship between AL, employees’ innovative performance, and aspects of CP [24, 38, 44, 45]. For example, in their recent work, Usman, Ghani [24] examined the relationship between AL and innovative work behavior among employees in the telecom sector in Pakistan. They found that AL sparks employee innovative work behavior, although they encouraged further studies. Furthermore, a study by Tung [38] exploring the role of AL on employee creativity among Chinese employees in the electronics sector, found that it significantly affected employee creativity, and encouraged further empirical studies. As mentioned above, and in line with previous research, CP in this study reflects both the cognitive elements of creativity and behavioral elements of innovative behavior [11]. A recent study by Saeed, Som [46] explored the influence of transformational leadership and AL styles on public hospital innovation and found that AL was a significant predictor. They encouraged further research on AL in health services research.

While studies in various contexts suggest a positive relationship between AL and aspects of CP [40, 47, 48], it is crucial to investigate this relationship in the health care context. Health care organizations often face distinct challenges, including stringent regulations, patient-centric care, interdisciplinary teams, resource constraints, patient diversity, risk aversion, evidence-based practices, technological advances, and complex processes [49–51]. These factors can influence how AL relates to CP among frontline health professionals [16]. Therefore, our study seeks to fill this research gap by examining the specific dynamics of the health care setting and knowledge of AL in health services research by examining its relationship with CP. Thus, to the best of the authors’ knowledge, this is a pioneering study of the relationship of AL with CP in a health services context.

Based on the discussion of theory and the literature review of previous empirical studies, there are good reasons to expect a similar pattern; that is, the practices of AL will be positively associated with CP among health professionals. The above reasoning leads to the following first hypothesis:

##### Hypothesis 1

There is a positive relationship between AL and CP.

#### Process mediators

Figure 1 suggests that LO and RL are process mediators between AL and CP. The two have one characteristic in common: they both focus on learning. Three reasons explain why learning as a concept is included in both LO and RL. First, learning is related to aspects embedded in CP, such as innovative behavior. The core of innovation is the introduction of changes and learning that increase the possibility of change through CP or innovative performance. Because of the important role of learning, Avby and Kjellström [12] described it as a key concept. Second, learning is intricately linked to leadership. It is well known that a leadership style can stimulate and strengthen learning in organizations in multiple ways and ideally foster what is labeled a “learning organization” [52]. Third, and this is a vital argument, learning is often considered one of the most critical sources of competitive advantage at the organizational level [52]. Consequently, there are good reasons to include and study learning as a process mediator for AL and CP.

Although LO and RL have a common focus on learning, the two differ considerably in how learning is manifested in the organization. Specifically, LO focuses on learning from an individual perspective. In contrast, RL centers on learning from a global and organizational perspective. The conceptual model presented in Fig. 1 assumes that AL not only correlates with the CP of health professionals, but it may also correlate with both LO and RL, which are process mediators between AL and CP. Consequently, the model assumes that exercising AL in organizations indirectly impacts the CP of health professionals. The form of the two process mediators and their associations with AL and CP are elaborated in detail below.

#### Learning orientation (LO)

LO refers to an employee’s self-engagement in various work tasks and situations [53]. Employees in organizations that focus on learning often exhibit high levels of problem-solving skills and commitment to learning new things and sharing knowledge in their work roles [54]. LO among employees has also been found to offer an organizational competitive advantage [53–55]. Continuous development through learning is an essential prerequisite for employees’ work success and continued organizational effectiveness [56]. This need for continuous development means that employees should have an attitude of engagement in and commitment to learning activities, a shared vision, be open-minded, and share organizational knowledge [54]. Engagement in learning activities is necessary and helps employees continuously update their skills and knowledge [57]. In this study, LO focuses on employees developing new knowledge and insights [58].

However, it is important to note that LO does not mean simply learning new knowledge and increasing one’s insight, as reflected in someone saying, “It is so exciting and enjoyable to learn something new occasionally.” Rather, LO is about learning with a potential benefit, goal, and purpose. Thus, LO refers to goal-oriented learning activities by employees. An orientation toward learning new knowledge or insights should potentially influence employee behavior [58]. This implies that LO is an active rather than passive search for ways to improve one’s knowledge and insights to benefit one’s work. As such, LO sometimes “involves questioning organizational practices and assumptions” [59]. For these reasons, the LO concept can be defined as an employee’s internal mind-set that motivates them to develop their competence [60]. Four important aspects characterize an employee’s LO.

First, LO is the cognitive state of a person, not of a group, team, or organization. LO is a personal orientation toward learning. Second, a person’s LO has a motivational aspect or inner drive. As the definition states, this primarily stems from a personal internal attitudinal mind-set that is comparable to an organizational commitment to learning [61]. Third, the definition of LO does not stipulate where learning occurs, its source, or whether it is formal (e.g., training or educational program, internal or external course/workshop) or informal (e.g., learning directly from one’s work). Because LO is an internal mind-set it will motivate or drive an individual to seek learning or knowledge actively, independent of where such opportunities arise. As Liu and Xiang noted, employees with higher levels of LO are more likely to engage in developmental activities [56]. Consequently, LO provides learning opportunities everywhere, both inside and outside the organization. Fourth, LO is a dynamic state instead of a (fixed) trait. Therefore, there may be opportunities to manage the LO of employees. This latter aspect is especially interesting and important, given the focus of this study, as it indicates that LO can be cultivated, shaped, and developed in the desired direction of organizational leaders (e.g., AL).

Scholars have long acknowledged that the style and practices of leaders in health service organizations are strongly related to multiple aspects of their employees, such as attitudes, motivations, and behavior [5, 23, 62–64]. Consequently, because of their position and formal authority and as role models, leaders possess the necessary tools to direct their employees in a preferred direction. Managers in committed organizations expect employees to use company time to pursue knowledge outside the immediate scope of their work. Health organizations that fail to encourage knowledge development have also been unable to motivate their employees to pursue learning activities. According to Ro, Yoo [53], LO depends on the value the organization assigns to learning. Where leaders practice AL, this implies that the organization values learning and growth [65]. As mentioned above, the goal of practicing open and closed leadership as a part of AL is to encourage explorative and exploitative behaviors [21]. In the literature, explorative and exploitative activities are closely associated with organizational learning [37]. Therefore, in line with Alghamdi [66], activities associated with exploitation and exploration behaviors are related to learning. Thus, through appropriate open and closed leadership practices, AL is designed to strengthen employees’ motivation and attitudes toward LO. Thus, it is proposed that the level of LO among individual employees is partly derived from positive employee perceptions of AL. To the authors’ knowledge, previous research linking AL to LO in health services research is scarce. However, previous research in other fields has found that leadership supports organizational learning [52, 67]. Clearly, employees’ perceptions of AL practices range on a continuum from highly positive to highly negative. However, based on the discussion above, there are good reasons to expect that when employees have a favorable impression of AL in their organization, it will strengthen their LO.

Learning Orientation (LO) is rooted in individuals’ intrinsic motivation to actively seek knowledge and insights for the purpose of improving their work. AL, with its dual focus on exploration and exploitation, aligns with the psychological underpinnings of LO. First, LO, as a cognitive state, is driven by an individual’s internal motivation and attitude [68]. AL, by encouraging both explorative and exploitative behaviors, provides a framework that resonates with employees’ innate drive for continuous development. Second, AL, through its open and closed leadership practices, sets the stage for goal-oriented learning. LO, in this context, becomes an active pursuit of knowledge with a specific purpose and benefit, mirroring the goal-oriented nature of AL. Third, Both AL and LO are dynamic states rather than fixed traits. This implies that, under the influence of AL, employees’ LO can be cultivated and developed in the desired direction, emphasizing the malleability of these psychological constructs. This leads to the second hypothesis:

##### Hypothesis 2

There is a positive relationship between AL and LO.

Previous research has found that leadership influences employees’ motivations, attitudes (cognition) as well as behavior (acts) [5, 23, 62–64]. Based on this simultaneous influence, AL and LO are expected to be associated with the CP of health professionals. Previous research has found that the level of an employee’s LO is associated with the ability to (think) creatively [60] and (act) innovatively [69, 70]. Organizations must be reinvented as creative powerhouses with a higher mission to move creativity into innovation to satisfy the end user’s needs and expectations. In this study, these two capabilities are reflected in CP.

However, it is assumed that leadership practices play a primary role as initiators of the above chain of associations. The fundamental premise is that AL can (kick)start this domino effect and, through its impact on employees’ LO, promote positive changes in employees’ CP capacity. Specifically, a shift exhibited in an improvement in AL practices (open and closed leadership practices) positively stimulates the LO of employees. It strengthens the development of new knowledge and insights [58] that are potentially beneficial to their performance. Finally, a positive shift in employees’ LO, derived from AL, may increase employees’ CP capability and ability to (think) creatively and (act) innovatively at work.

Consequently, this reasoning considers CP to be a function of learning and knowledge development [12] reflected in the level of LO resulting from the quality of organizational AL. LO contributes to explaining the process (what really happens) and is a necessary mediating factor in the relationship between AL and CP. To the authors’ knowledge, this is a pioneering study in health services research examining employee LO as a mediator between AL and employee CP. However, findings in other settings indicate such a chain of links starting from organizational leadership practices. For example, in a study by Mutonyi, Slåtten [70], the authors found that the individual LO of public sector employees (in a transportation company) mediated the relationship between empowering leadership practices and employees’ innovative behavior. Similarly, the current study proposes that employee LO mediates between AL practices and employee CP at work. Based on this reasoning and the discussion above, the third hypothesis is:

##### Hypothesis 3

The relationship between AL and CP is mediated by LO.

#### Relationship learning (RL)

Regarding health professionals, Li, Grimshaw [71] argued that interacting with peers in the workplace (e.g., other health professionals) fosters learning and knowledge sharing. This interaction represents RL in this study. Compared with the concept of LO, which has a narrow focus on individual employees’ development of new knowledge and insights [58], RL takes a significantly broader perspective and embraces an organizational focus on learning. However, RL is not as broad as organizational learning, which is the whole organization’s learning capability. In contrast, RL embraces learning within a community in an organization. A community of practice is where learning occurs [72].

Specifically, RL is reflected in interplay with colleagues in similar roles. Organizations can promote RL by cultivating a collaborative culture, formulating specific objectives for joint learning activities, and developing relational trust [73]. According to Li, Grimshaw [71], communities provide a safe environment where employees can freely learn through observation and interaction with colleagues and other experts. Consequently, RL refers to a community of (similar) practice capability from which people learn, reflected in such learning activities as sharing, discussing, and assessing information potentially beneficial to work performance. Thus, RL can be described as reflecting the learning climate (how things are done here) in an organizational community of practice to improve organizational performance, always based on the concerns or passions of these voluntary groups.

There are two important aspects regarding perceptions of RL in this study. First, RL is considered a climatic state of the community, not a fixed trait. RL is assumed to be changeable, positively or negatively, over time. The reasons for such changes in RL could be new members entering the community of practice or a new leadership style that strengthens or weakens the RL climate. Second, it is important to note that RL indicates nothing specific about whether learning is planned, organized, ad hoc, or a mix of informal and formal learning activities. This latter aspect reflects the choice of this study to focus on a holistic view of RL and health professional employees’ perceptions of the learning activities (or RL climate) within their community of practice.

Leadership is an essential tool to shape and direct the organization. Because of their authority, leaders’ potential is not limited to their impact on individual employees; they also impact communities of employees. Leaders can stimulate the learning attitudes, motivation, and behavior of members of a community of practice. Previous research has found leadership practice can promote and stimulate members to become a learning organization [52]. Because the practice of a leader impacts the learning in the entire organization, there are reasons to suggest that these also apply to other areas of an organization, such as communities of practice. Thus, in health care, communities of practice are seen as a tool to enhance knowledge and improve practice [72, 74, 75].

In this study, it is assumed that AL is related to RL. As mentioned above, the theory of AL was originally designed specifically to improve the potential for innovation in an organization [36]. However, innovation is an objective of learning and knowledge creation integrated into daily work tasks [12]. Thus, AL implicitly values the desire and need for learning. As learning is a prerequisite for innovation, leaders practicing it should be strongly motivated to stimulate the RL climate in communities of practice. Accordingly, AL manifested in a combination of open and closed leadership practices would advance employees’ explorative and exploitative behaviors [21]. In turn, this would promote learning in an organization [37].

To the authors’ knowledge, this is a pioneering empirical study in health services research exploring the relationship between AL and RL. Previous research in other fields has found that leadership practices are associated with learning in the entire organization [52] and communities of practice, such as professional service teams [76]. In line with this, Marsick [77] maintained that leadership influences the work climate for learning. Therefore, this study proposes that AL positively correlates with RL, through which collaborative learning occurs [78].

RL, as reflected in interactions within communities of practice, is influenced by leaders who shape the organizational climate for learning. AL, designed to enhance innovation, implicitly values and encourages learning. The psychological link between AL and RL can be explained as follows: First, by the leadership impact on Learning Attitudes. Leaders, especially those practicing AL, have a significant impact on the learning attitudes, motivations, and behaviors within communities of practice. AL’s original objective of improving innovation aligns with the collaborative learning environment fostered by RL [77, 79]. Second, AL, through its promotion of both exploration and exploitation, inherently values learning as a prerequisite for innovation. RL, focusing on learning within a community, becomes a natural outcome of leaders encouraging explorative and exploitative behaviors. In fact, AL’s orientation towards innovation implies a strong motivation to stimulate RL within communities of practice. The positive correlation between AL and RL suggests that the practices of AL leaders contribute to a conducive climate for collaborative learning, enhancing knowledge sharing and performance improvement within specific organizational communities. In sum, these reasoning leads to the fourth hypothesis in this study:

##### Hypothesis 4

There is a positive relationship between AL and RL.

In a continuation of Hypothesis 4, and as presented in the conceptual model (see Fig. 1), the study proposes that AL has both a direct and an indirect correlation with RL. Specifically, the study proposes that AL, through its impact on RL, also affects employees’ CP. In this study, CP reflects a combination of both creativity and innovative behavior [11].

Encouraging employees to contribute their unique creative outputs to an organization can be a pivotal source of innovation and continuous organizational growth. Cognitive diversity has been demonstrated to increase group creativity and is related to CP. Consequently, when there is RL among the community of practice members, manifested in a positive and stimulating learning environment, it should be a resource to develop new ideas (think creatively) and implement them (act innovatively) to improve members’ work performance.

From the role of AL, it is assumed that employee CP is a function of learning and knowledge creation [12], reflected in the level of RL that stems from AL practices. RL is a mediating process and reflects the actions of a community of practice, which may explain the relationship between AL and employee CP. Thus, there are good reasons to expect RL to mediate between AL and CP. This assumption about the pattern of relationships is summarized in the fifth and final hypothesis:

##### Hypothesis 5

The relationship between AL and CP is mediated by RL.

To summarize, the current study proposes the following hypotheses based on the conceptual model of the study (see Fig. 1):

##### Hypothesis 1

There is a positive relationship between AL and CP.

##### Hypothesis 2

There is a positive relationship between AL and LO.

##### Hypothesis 3

The relationship between AL and CP is mediated by LO.

##### Hypothesis 4

There is a positive relationship between AL and RL.

##### Hypothesis 5

The relationship between AL and CP is mediated by RL.

## Methods

### Data collection procedure

This study aims to examine empirically the role of AL in facilitating health professionals’ CP. Previous health services research has emphasized the importance of gaining knowledge on AL and its potential influence on CP [39]. Nevertheless, few empirical studies extend the theoretical foundations of AL. Consequently, this study explores AL by sampling Norwegian health professionals in nine homecare service institutions in a medium-sized city in Norway. This responds to a call for further knowledge on homecare and patient needs [80]. Consistent with Hewko [13] and Grasmo, Liaset [81], health professionals in homecare service institutions are understood to be individuals who have obtained a university degree in a relevant subject or have received health aide certification.

All contact with the homecare service institutions and their health professionals was sought through the Director of Knowledge and Development (DKD). Several meetings were held to introduce, explain, and recruit for the intended study. Note that the current study is part of a larger research project. Furthermore, to minimize potential influences on respondents [81], all communication and information regarding the study went through the DKD, who forwarded the information to the department heads, who then sent it to their employees. The information described the study project and its purpose, explained that participation was voluntary and anonymous, and sought consent. This process was repeated during the pretest on the survey quality, the final survey link, and a reminder to participate. Three experts in the field and a few health professionals completed a pretest to ensure readability, conducted back-to-back translation (English–Norwegian), and offered areas for improvement. The final survey was accessed through the *Nettskjema* platform (www.nettskjema.no), which offers complete anonymity, including automatic deletion of IP addresses upon survey completion. Consent was ensured by participants clicking on “Next.” To comply with Norwegian personal data protection regulations, the complete survey was sent to the Norwegian Centre for Research Data (NSD/SIKT) for approval.

Approximately 500 health professionals from nine homecare service institutions were invited to participate in the survey; *N =* 258 completed the survey, a 61.6% response rate. The personal characteristics of those in the study sample are summarized in Table 1.

**Table 1:** Personal characteristics of the study participants (*N* = 258)

		%
Staff role	Nurse	34.5
	Health professional	49.2
	Other (health professionals (bachelor’s, unskilled))	16.3
Employed	< 5 years	35.7
	6–15 years	26.7
	16–25 years	23.3
	> 25 years	14.3
Work hours	Part-time	66.7
	Full-time	33.3
Age	< 35 years	27.5
	35–50 years	38.4
	> 50 years	34.1

### Instruments

The importance of the health professionals’ perspective in exploring the role of AL in CP and its process mediators is consistent with previous research [5–7, 15]. Furthermore, previous research calls for further knowledge, and despite the dearth of research on the factors proposed in the conceptual model of this study (see Fig. 1), AL, CP, LO, and RL all have scales based on well-established theories. The items used for this study are from existing scales adapted for the study context: homecare service institutions. Adaptations included language and cultural context owing to the geographical boundaries of Norway. Each item was measured using a seven-point Likert scale, ranging from “strongly disagree” [1] to “strongly agree” [7]. The survey included demographic characteristics (Table 1). AL was adapted and measured through a 10-item scale from Rosing, Frese [21] and Zacher, Robinson [36]. AL in this study consists of two constructs: leaders’ opening and closing behavior. As recommended by Rosing, Frese [21] and Zacher, Robinson [36], AL should be studied in its entirety and not as a second-order construct, as the definition of AL includes both opening and closing behavior. *Learning orientation (LO)* was adapted and measured using a three-item scale from Mutonyi, Slåtten [70] and Sujan, Weitz [82]. *CP* was adapted and measured using a seven-item scale from Mutonyi, Slåtten [83]. *Relationship learning (RL)* was adapted and measured using a five-item scale from Slåtten, Lien [76]. The complete list of the constructs (AL, CP, LO, and RL) and claims are summarized in Table 2. Note that the claims used in this study are part of a larger research project.

**Table 2:** Constructs and items used in the study

Construct	Item label	Items
Ambidextrous leadership (AL)		
AL	AL1	Allows different ways of accomplishing tasks.
	AL2	Encourages experimentation with different ideas.
	AL3	Gives opportunities for independent thinking and acting.
	AL4	Allows room for colleagues’ ideas.
	AL5	Allows errors.
	AL6	Encourages learning from errors.
	AL7	Monitors and controls goal attainment.
	AL8	Establishes routines.
	AL9	Takes corrective action, if necessary.
	AL10	Controls adherence to rules.
	AL11	Sticks to plans.
Creative performance (CP)		
CP	CP1	Creates new ideas to solve problems.
	CP2	Searches out new working methods to improve own job performance.
	CP3	Investigates and finds ways to implement own ideas.
	CP4	Promotes own ideas so others might use them in their work.
	CP5	Tries out new ideas in own work.
	CP6	Comes up with creative solutions to problems.
	CP7	Suggests new ways to increase quality.
Learning orientation (LO)		
LO	LO1	Acquires new knowledge when necessary.
	LO2	Feels it is worth spending a great deal of time learning new ways to accomplish own work.
	LO3	Feels it is important to continually improve own professional skills.
Relationship learning (RL)		
RL	RL1	Team colleagues exchange information about successful and unsuccessful experiences with their own services.
	RL2	Colleagues in own team exchange information related to changes in end-users’/patients’ needs.
	RL3	Colleagues in own team exchange information as soon as possible if any unexpected problems occur.
	RL4	Colleagues in own team stimulate discussion, encompassing a variety of opinions and thoughts.
	RL5	Colleagues in own team frequently evaluate and update information stored in different databases.

### Data analysis

To test the hypotheses in our conceptual model, we employed partial least-squares structural equation modeling (PLS-SEM) [84] as our data analytical procedure, using SmartPLS 3 software [85]. PLS-SEM is based on a two-stage approach. The first stage assesses the measurement models, while the second assesses the structural model. Based on the PLS-SEM results, mediator effects are estimated and analyzed using the bootstrapping test of Zhao, Lynch [86].

## Results

### Measurement models

In our study, the measurement models contained only reflective constructs. The assessment was based on (i) convergent validity (the extent to which a variable is positively correlated with alternative variables used to measure the same construct, i.e., loading and average variance extracted); (ii) internal consistency reliability (the magnitudes of the intercorrelations of the observed variables, using the criteria of composite reliability and Cronbach’s alpha); and (iii) discriminant validity (the extent to which a construct is distinct from other constructs, using the heterotrait–monotrait ratio criterion). We used the “rules of thumb” criteria of Hair, Hult [84] to measure the quality of the constructs. The results shown in Table 3 indicate that we have reliable and valid measurement models.

**Table 3:** Results of the measurement model for the AL, CP, LO, and RL constructs

		Convergent validity		Internal consistency reliability		Discriminant validity
Construct	Claims label	Indicator reliability	AVE^*^	Composite reliability	Cronbach’s alpha	HTMT criterion^*^
Rules of thumb		Loading > 0.7	> 0.5	0.7–0.95	0.7–0.95	HTMT interval does not include 1
AL	AL1	0.80	0.60	0.94	0.93	Yes
	AL2	0.84				
	AL3	0.70				
	AL4	0.86				
	AL5	0.69				
	AL6	0.81				
	AL7	0.67				
	AL8	0.81				
	AL9	0.84				
	AL10	0.77				
	AL11	0.71				
CP	CP1	0.75	0.61	0.92	0.90	Yes
	CP2	0.79				
	CP3	0.81				
	CP4	0.79				
	CP5	0.82				
	CP6	0.74				
	CP7	0.78				
LO	LO1	0.87	0.67	0.86	0.76	Yes
	LO2	0.81				
	LO3	0.78				
RL	RL1	0.83	0.72	0.93	0.90	Yes
	RL2	0.88				
	RL3	0.90				
	RL4	0.84				
	RL5	0.77				

^*^ AVE = Average variance extracted; HTMT = Heterotrait–monotrait ratio of correlations

### Structural models

**Fig. 2:**
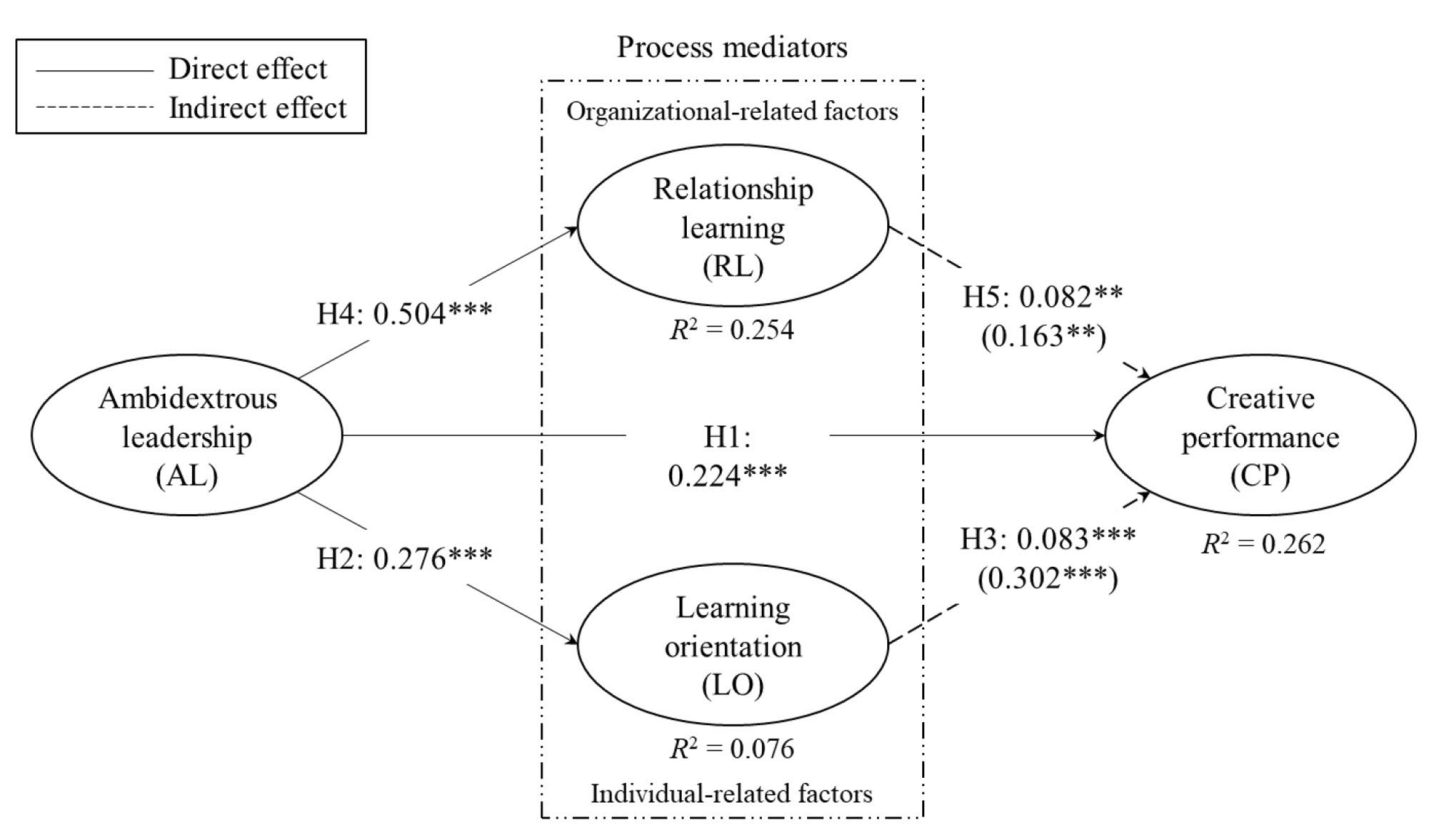
Direct and indirect effects in the structural model linking AL, CP, LO, and RL. Standardized coefficients (*** < 0.01, ** < 0.05). Note that the reported path parameters on the dotted lines are indirect, while the direct effects are shown in parentheses.^1^

The results of the structural model are presented in Fig. 2. Before the structural model is assessed, it is important to ensure that there is no collinearity issue in the structural model. The highest value inflaction factor (VIF) value is 1.4. Following the reccomendations of Kock [87] in testing for common method bias in PLS-SEM models, our VIF of 1.4 is an indication that the model is free of common method bias. In addition, all constructs are below the rule-of-thumb value of 3 suggested by Hair, Hult [84]. For the endogenous constructs, the in-sample predictive power of the model (*R*^2^) was 0.262 for CP and 0.254 for RL. Acceptable *R*^2^ values are based on context [84]; however, we consider these *R*^2^ values moderate. For LO, *R*^2^ was 0.076.

Health professionals’ AL significantly affects their CP, which supports Hypothesis 1. Hypothesis 2 is also supported, indicating a positive relationship between AL and LO. Moreover, we find a significant positive association between AL and RL, which supports Hypothesis 4.

Hypotheses 3 and 5 concern mediator effects, and we find support for both. This means that the relationship for health professionals between AL and CP is mediated by both LO (Hypothesis 3) and RL (Hypothesis 5).

## Discussion

Recent research in health services has called for further knowledge of the potential of various leadership styles for leading innovation [23]. This study answers this call by exploring AL because it has previously been suggested that this style has components that facilitate CP among health professionals. The results of this study reveal that when health professionals are given flexibility within a set framework, their CP, LO, and RL are linked.

In short, the purpose of this study has been to explore empirically factors that influence health professionals’ ability to think creatively and act innovatively, namely CP. There are three main contributions. First, the study contributes to our current understanding of the relationship between AL, CP, LO, and RL. Second, by extending previous research on AL in health services research, we answer the call for more research on factors related to CP. Third, we deepen our insights into the role of learning and flexibility within a set framework for encouraging innovation among health professionals and their work roles.

The study’s conceptual model (Fig. 1) suggests five hypotheses: three direct relationships and two indirect or mediating ones. The results of this study, shown in Fig. 2, support the five (H1–H5) hypothesized relationships in this study. Specifically, AL is positively associated with CP, LO, and RL. In addition, the findings confirm that LO and RL partially mediate the relationship between AL and CP.

Though our findings coincide with previous studies that have found positive links between AL and aspects of CP [24, 36, 88], the findings and contributions of this study differ in three ways. First, the findings of prior studies focused on factors that have aspects of CP, such as team innovation [36], innovative work behavior [24], employee creativity [38], and employee innovation [88]. Second, prior studies were often conducted in other research domains, such as the telecom sector [89], public museums [88], architectural and interior design firms [36], and electronic companies [38]. Our findings contribute new knowledge to the ongoing theoretical debate over the role of AL in employee CP. Furthermore, previous research considers the complex and nonlinear nature of AL and innovation [21, 30], where managers must balance between fostering employees’ explorative and exploitative behaviors and allowing them to challenge the status quo by exploring new ideas and implementing them at work. In line with this, Anderson, Potočnik [30] and Usman, Ghani [24] asserted that AL is the most suitable leadership approach to address this complexity. Third, previous studies on the relationship between AL and aspects of CP have mostly concerned the two components separately. This study has composed CP from creativity and innovative behavior, as is appropriate for the complex nature of AL, which encourages employees’ creative and innovative behavior. Our findings reveal that the flexibility of AL [21], balancing and combining opening and closing behavior, is positively related to health professionals’ CP.

Our findings are consistent with AL theory, which emphasizes that employee CP is highest when leaders exercise high levels of opening and closing behavior [39]. This is also evident in the results of this study (see Fig. 2), where AL explains about 26% (R^2^ = 0.262) of the variance of CP, indicating a substantial capability to drive it. Consequently, our discoveries strengthen the previous studies in empirically exploring AL in a health sector setting, renowned for its intricacy, and offer new insights into the complex relationship of AL and CP in the professional setting [24, 36, 38, 39].

Moreover, the results of our study demonstrate a significant positive relationship between AL and LO. Previous studies have found a positive association between AL and organizational learning [37]. Though Huang and Li [90] examined AL as a mediating factor between LO and product performance, the findings of their study support ours. However, they differ regarding the direct and indirect relationships. For example, previous research has often studied LO either as an independent factor or as a mediator [60, 70, 90, 91]. The findings of this study offer new knowledge on LO as a dependent factor and strengthen the current theoretical discourse on a leader’s role in supporting learning at work. Accordingly, previous research has argued that LO encompasses learning for a purpose [58, 70]. Specifically, for health professionals, this entails actively seeking and improving one’s knowledge and competence to benefit patients, oneself, and the organization [60]. This means that a leadership style such as AL also facilitates health professionals’ LO, which can improve an employee’s search for and acquisition and use of new knowledge to work more efficiently.

Additionally, the results of this study offer empirical evidence and validate the view that AL is positively and significantly associated with health professionals’ RL. Our study’s results align with previous findings that revealed various leadership practices to be positively related to LO [52] and RL [76]. However, the results of our study on the relationship between AL and RL contribute new knowledge in two ways. First, our findings make a unique contribution to the learning theory by expanding the empirical study of RL, as suggested by Slåtten, Lien [76], in addition to broadening leadership practices such as AL and their role in employee RL, as suggested by Delić, Slåtten [52]. Second, our results add to previous findings on contextual aspects. Specifically, the current study focuses on health professionals in homecare service institutions, contributing further understanding of the relationship between AL and RL in a health sector setting. This is especially crucial as previous research has asserted that leadership generally strongly influences the climate of learning at work [77], which in turn affects innovative employee capabilities [12]. Interestingly, this is also reflected in our results (see Fig. 2), where AL explains about 25% (R^2^ = 0.254) of the variance of RL, indicating a substantial influence on health professionals’ RL. Our findings, therefore, strengthen previous studies in empirically exploring AL in a health sector setting known for its complexity. In addition, they offer new insights into a generally unexplored yet important relationship between AL and RL at work [12, 52, 67, 76].

The conceptual model of this study (see Fig. 1) proposed LO and RL as process mediators between AL and CP. The findings of our study (see Fig. 2) offered empirical evidence and validated the role of LO and RL as intervening mechanisms connecting AL to CP. Specifically, the study found that LO and RL mediate the relationship between AL and CP, which aligns with previous research findings, albeit with minor differences. In detail, previous research has indicated support for learning, where it was found that individual LO mediated the relationship between empowering leadership and employee innovative behavior [70]. Therefore, the results of this study confirm that positive shifts in health professionals’ LO and RL that stem from AL are positive “ingredients” of health professionals’ capability to think creatively and act innovatively.

In summary, the findings of this study offer new evidence that AL fosters explorative and exploitive behaviors among health professionals [21], ensuring their LO and RL, which in turn lead to higher levels of CP. Therefore, there are several practical implications from this study.

### Practical implications

The theoretical implications of this study offer new insights that suggest three practical implications for health managers.

First, as mentioned above, AL draws on four distinct states: goal and purpose, process of practice, flexibility, and ability. Therefore, the results of this study suggest that health managers play a crucial role in fostering AL qualities that positively influence their employees. Provided that AL is a state of a person, and it can be taught and training given, ambidextrous health managers who portray AL at work will help to nurture a work environment that encourages health professionals to exhibit higher levels of CP. For example, health managers may facilitate discussions highlighting the importance of allowing errors and independent thinking, encouraging experimentation, and making room for novel ideas. Similarly, health managers should apply AL behaviors that establish equal and clear routines and adherence to rules while monitoring goal attainment to ensure that communities of practice collaborate to achieve organizational goals. This way, health managers will cultivate an innovative environment promoting CP.

Second, today’s unpredictable environment has highlighted the importance of fostering employee learning in increasing firms’ innovation capabilities and performance [54]. Health professionals exist in unique working environments, such as homecare service institutions, which require continuous updating of skills and knowledge [57]. The results of this study underscore the importance of the study by Liu and Xiang [56], who maintained that employees with high levels of LO often engage in innovative activities, such as investing in ways to implement novel ideas at work. Health managers should thus facilitate a learning environment where health professionals perceive it as being worthwhile to spend time acquiring new knowledge to accomplish their work tasks or improve their professional skills. This should spill over to their RL, where their communities of practice will feel motivated and unrestricted in exchanging information related to patient needs, and they will evaluate and/or update important information that will improve service at homecare institutions. Adjusting work environments to encourage learning is important for individual health professionals and teams, as it creates an atmosphere that stimulates discussion encompassing a variety of opinions.

Third, the results of this study demonstrate the value of process mediators—LO and RL—in the relationship between AL and CP. Specifically, the findings of our study suggest that health managers are in a compelling position to develop a work environment where employees can learn individually and in a team, simultaneously encouraging more CP. Prior studies by Barr and Dowding [92] and Jensen [93] have emphasized the value of leadership behaviors in promoting and cultivating health employees’ creative and innovative behaviors, or CP. Moreover, Ghoshal, Bartlett [94] recognized the key value of AL, namely that it fosters synergy between employee exploration and exploitation. This means that in our findings, AL can boost health professionals’ CP, LO, and RL. Therefore, by drawing on the four distinct features of AL, namely goal and purpose, process of practice, flexibility, and ability, health managers are encouraged to train themselves in AL components, which will promote health professionals’ LO and RL. This, in turn, will facilitate health professionals’ CP.

### Limitations and future research

While the theoretical foundations and the practical implications of this study offer various meaningful contributions to knowledge on AL, CP, LO, and RL, it also has limitations. To the best of the authors’ knowledge, this is a pioneering empirical study of the direct and indirect relationships of AL, CP, LO, and RL in a health services research setting. Three main limitations offer opportunities for future research.

First, the present study employed convenience sampling, limiting the data collection from one health organization with its nine homecare service institutions. As the focus was on health professionals in Norwegian homecare service institutions, the findings of this study cannot be generalized, as is evident from its cross-sectional nature. In addition, convenience sampling combined with online surveys entails issues of self-selection bias and possible reversed causality. Furthermore, although this study followed the recommendations of Kock [87] and Hair, Hult [84] in testing for common method bias, the study´s results migh still suffer from common method bias errors [95]. Consequently, future research could compare its findings with population data, adopt other means of data collection, and explore discrepancies in a variety of health institutions or departments. For example, Tung [38] noted that qualitative studies with in-depth interviews of participants may offer different insights.

Second, this study focused on the direct and indirect relationships between four factors—AL, CP, LO, and RL—among health professionals. Future research could further the theoretical insights and debate by assessing other leadership variables that may be important in facilitating health professionals’ CP. For instance, future research could examine how leadership behaviors impede or foster health professionals’ work curiosity [96].

Third, this study examined two process mediators: LO and RL. The study offers a unique understanding on which future research can draw. Specifically, as learning has become vital in the modern transitional economy [52], the need to further current knowledge on the role of leadership behavior in cultivating learning environments is of the essence [34, 45, 56]. In particular, future research can explore other process mediators such as thriving at work [97], employee attractiveness [5], service quality of care [98], and work performance [58].

In summary, Zacher, Robinson [36] provided “a useful first step concerning the evaluation of the value of the ambidexterity theory of leadership for innovation” (p. 43). The results of this study may be a stepping stone to a much larger discussion exploring leadership behavior in health service organizations.

## Data Availability

The data sets used and/or analyzed in this study are available from the corresponding author upon reasonable request.

## Footnotes

1 To save space, we did not report all estimated parameters in the structural model or the mediator analysis. However, these results are available from the authors upon request

## Abbreviations

SEM: Structural Equation Modeling
PLS-SEM: Partial Least Structural Equation Modeling
SRMR: Standardized Root Mean Square Residual
RMSEA: Root Mean Square Error of Approximation
HTMT: Heterotrait–Monotrait Ratio of Correlations
CFI: Comparative Fit Index
TLI: Tucker–Lewis Index
RCC: Raykov’s Reliability Coefficient
AVE: Average Variance Extracted
VIF: Variance Inflation Factor
SQ: Service Quality
NSD/SIKT: Norwegian Social Science Data Services
DKD: Director of Knowledge and Development
CP: Creative Performance
AL: Ambidextrous Leadership
LO: Learning Orientation
RL: Relationship Learning
IWB: Innovative Work Behavior
